# Phylodynamics of HIV-1 in Lymphoid and Non-Lymphoid Tissues Reveals a Central Role for the Thymus in Emergence of CXCR4-Using Quasispecies

**DOI:** 10.1371/journal.pone.0000950

**Published:** 2007-09-26

**Authors:** Marco Salemi, Brant R. Burkhardt, Rebecca R. Gray, Guity Ghaffari, John W. Sleasman, Maureen M. Goodenow

**Affiliations:** 1 Department of Pathology, Immunology, and Laboratory Medicine, University of Florida, Gainesville, Florida, United States of America; 2 Department of Anthropology, University of Florida, Gainesville, Florida, United States of America; 3 Department of Pediatrics, Division of Immunology, Rheumatology, and Infectious Diseases, University of Florida, Gainesville, Florida, United States of America; 4 Department of Pediatrics, Division of Allergy, Immunology, and Rheumatology, University of South Florida and All Children's Hospital, St. Petersburg, Florida, United States of America; National Institute for Communicable Diseases, South Africa

## Abstract

**Background:**

During HIV-1 infection coreceptor switch from CCR5- (R5)- to CXCR4 (X4)-using viruses is associated with disease progression. X4 strains of HIV-1 are highly cytopathic to immature thymocytes. Virtually no studies have evaluated the HIV-1 quasispecies present *in vivo* within thymic and lymphoid tissues or the evolutionary relationship between R5 and X4 viruses in tissues and peripheral blood.

**Methodology/Principal Findings:**

High-resolution phylodynamic analysis was applied to virus envelope quasispecies in longitudinal peripheral blood mononuclear cells (PBMCs) and lymphoid and non-lymphoid tissues collected *post mortem* from therapy naïve children with AIDS. There were three major findings. First, continued evolution of R5 viruses in PBMCs, spleen and lymph nodes involved multiple bottlenecks, independent of coreceptor switch, resulting in fitter quasispecies driven by positive selection. Second, evolution of X4 strains appeared to be a sequential process requiring the initial fixation of positively selected mutations in V1-V2 and C2 domains of R5 variants before the emergence of high charge V3 X4 variants. Third, R5 viruses persisted after the emergence of CXCR4-using strains, which were found predominantly but not exclusively in the thymus.

**Conclusions/Significance:**

Our data indicate that the evolution of X4 strains is a multi-step, temporally structured process and that the thymus may play an important role in the evolution/amplification of coreceptor variants. Development of new therapeutic protocols targeting virus in the thymus could be important to control HIV-1 infection prior to advanced disease.

## Introduction

Infection of target cells by human immunodeficiency virus type 1 (HIV-1) requires binding of the viral surface protein gp120 to the cellular surface protein CD4 and chemokine receptors CCR5 or CXCR4 [1]. R5 viruses using the CCR5 coreceptor represent the predominant viral quasispecies during the early and chronic phases of the infection [2], [3]. X4 viruses using the CXCR4 coreceptor appear at a later stage in about 50% of individuals infected by HIV-1 subtype B and are associated with accelerated disease progression [4], [5]. The reasons for coreceptor evolution during the course of infection and the origin and evolution of X4 strains are not fully understood, although several hypotheses have been proposed [6]. Appearance of X4 viruses might reflect emergence of quasispecies sequestered in tissues at the time of infection [7] or evolution *de novo* from R5 viruses [8]–[10].

The primary genetic determinants of HIV-1 coreceptor use are concentrated within the 35-amino acid hypervariable V3 loop of the envelope protein gp120 [11]–[13]. Although a small number of basic amino acid substitutions in V3 may be sufficient for changes in coreceptor preference, combinations of V3 mutations can lead to major loss of entry fitness in culture, unless compensated by mutations in or near V1-V2 in gp120 [14], indicating that complex, discontinuous determinants contribute to X4 coreceptor use, at least on certain cell types [9], [15], [16].

Continuing HIV-1 replication in anatomic or cellular reservoirs and release of latent virus from infected reservoirs can contribute to viral rebound following interruption of combination anti-retroviral therapy (ART) [17], [18]. Genital tissues and blood appear to serve as distinct reservoirs harboring latent HIV-1 during prolonged drug therapy [19], [20], while the brain is a viral compartment harboring HIV-1 subpopulations with specific genetic characteristics [21]–[26]. CD4 T lymphocytes in infants and children predominantly express CD45RA, whereas in adults about equal ratios of CD45RA or CD45RO are expressed [27]. Only a subset of activated CD4 CD45RO T cells express CCR5, while the preponderance of CD4 T-lymphocytes, independent of CD45 isoform, express CXCR4 coreceptors [27], [28]. The thymus harbors a large number of immature and mature CD4 thymocytes expressing CXCR4, but relatively limited CCR5-expressing cells, implicating the thymus as a critical compartment for HIV-1 pathogenesis [29]–[32]. X4 viral strains are highly cytopathic to immature thymocytes *ex vivo* [33]. Within HIV-1 infected individuals, significant reduction in thymocyte proliferation, output and function occurs in the absence of ART [34], [35], while HIV-induced destruction of the thymus decreases the capacity for T-cell immune reconstitution resulting in rapid disease progression in infected children [36]. Despite the importance of X4 strains for pathogenesis, virtually no studies have evaluated coreceptor use or the evolutionary patterns across hypervariable regions of HIV-1 *env* quasispecies infecting the thymus *in vivo* [37].

Recently, a “*phylodynamic*” framework using phylogeny and coalescence theory was developed and applied to study evolutionary dynamics of pathogens within infected hosts [26], [38]. In the present work, we applied high-resolution *phylodynamics* to analyze HIV-1 subpopulations (*virodemes*) infecting the thymus, lymphoid and non-lymphoid tissues that may act as viral compartments and/or reservoirs [24], and longitudinal peripheral blood mononuclear cells (PBMCs) from HIV-1 infected children. The goal was to track the tempo and mode of appearance of X4 strains *in vivo*, to investigate the role of the thymus, and to uncover the direction of viral gene flow among tissues.

## Results

### Characterization of HIV-1 viral quasispecies in tissues and peripheral blood

In each subject V3 amino acid residues revealed a mixture of sequences with low or high net charge predicting, as confirmed by two independent algorithms, CCR5 or CXCR4 coreceptor use respectively [39]–[41]. Three envelope sequences from the thymus, for which the two algorithms gave discordant results, were characterized by functional analysis with single-cycle, Env-pseudotyped viruses [9]. Two (from subject S1 and S2, respectively) used the CXCR4 coreceptor exclusively, while one (from subject S4) used both CCR5 and CXCR4 coreceptors.

**Figure 1:**
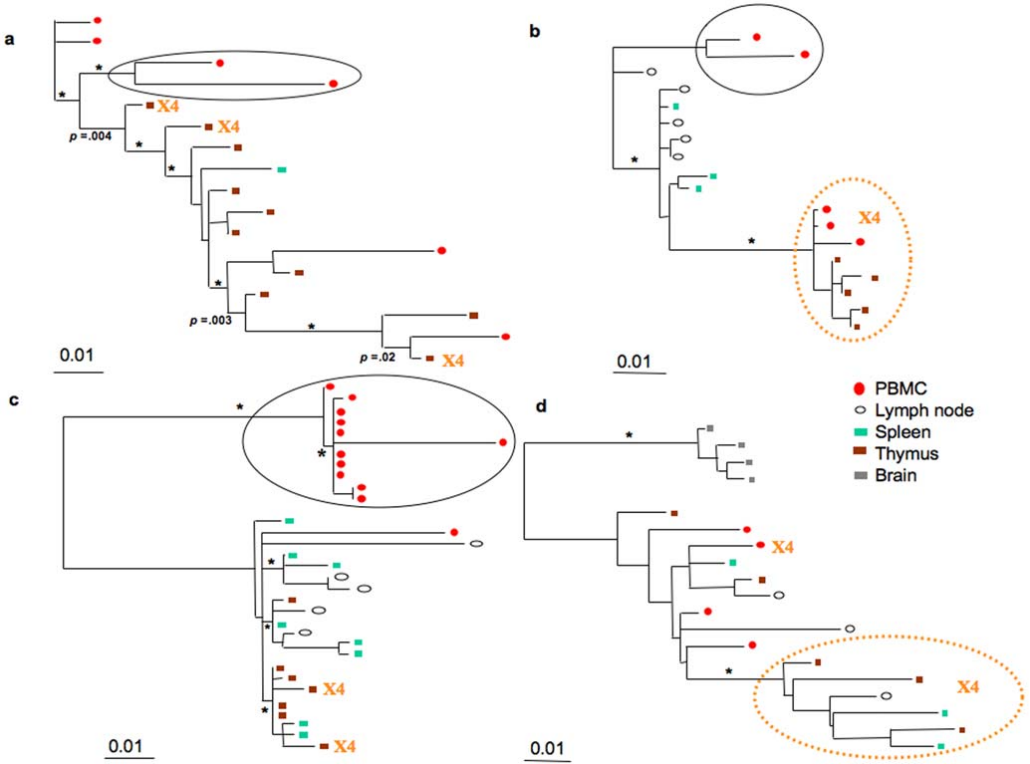
Maximum likelihood trees of HIV-1 V1-V3 sequences from *post mortem* tissues and PBMCs of different subjects. Branch lengths were estimated with the HKY+Γ model and were drawn in scale with the bar at the bottom indicating 0.01 nucleotide substitutions per site. The position of the root was inferred by maximum likelihood enforcing the molecular clock constraint. One * along a branch represent significant statistical support for the clade subtending that branch (zero-branch-length test *p*<0.001 and a bootstrap value >70%). Colored boxes and circles represent different tissues according to the color table in the figure. Monophyletic clades of PBMCs with R5 sequences are highlighted within solid circles. Broken circles highlight monophyletic clades of X4 sequences. a. Subject S1. b. Subject S2. c. Subject S3. d Subject S4.

Maximum likelihood (ML) phylogenetic trees estimated from the V1-V3 alignments of sequences sampled at the time of death from four subjects displayed significant branches among the quasispecies independent of length of infection (Figure 1). In subjects S1, S2 and S3 a well-supported subclade of R5-using viral variants was localized in PBMCs and distinct from quasispecies in contemporaneous tissues where X4 and R5 variants commingled (Fig. 1a, 1b, and 1c). X4-using strains were identified exclusively in the thymus from subjects S1 and S3, in thymus and PBMCs from subject S2, and in thymus, lymphoid tissues and peripheral lymphocytes from subject S4. Sporadic X4 strains were intermixed with R5 ones in patients S1 (Figure 1a) and S3 (Figure 1c). In contrast, a well-supported monophyletic clade of X4 strains emerged from an R5 population in patients S2 (Figure 1b) and S4 (Figure 1d). In all cases, X4 variants always clustered on branches that appeared to emerge from R5 ancestors. The tree inferred for sequences from each subject included at least one significantly supported branch within the R5 lineage, suggesting that emergence of successful viral subpopulations may require selective pressures not exclusively linked to evolution of V3 variants with altered coreceptor use.

### Analysis of recombinant sequences

HIV-1 frequently recombines *in vivo* [42]–[44]. Since intra-patient recombination would lead to the creation of mosaic genomes violating the tree-like assumption of evolution, we carefully checked for recombinant sequences within our data sets before performing high-resolution phylogenetic analysis. ML trees inferred from V1-V2 or C2-V3 domains were identical for S1 and S3, indicating no obvious intra-patient recombination. The finding was confirmed by the PHI test for recombination (*p*>0.05). In contrast, six sequences (10.9%) from S2 and 47 (31.3%) from S4 clustered in different clades depending on the domain used to infer the trees. Significant evidence of recombination was detected by the PHI test in both S2 and S4 alignments (*p*<10^−6^). When putative recombinant sequences were removed from S2 and S4 alignments, the PHI test was no longer significant (*p*>0.05). Recombinant sequences of R5 or X4 phenotype were predominantly detected in tissue samples rather than peripheral lymphocytes (Table S1). In subject S4 about two-thirds of the recombinant sequences were found in the brain. It is important to notice that the low rate of PCR-mediated recombination (<2% per 1000 nucleotides) [45], [46], and the significantly different distribution of recombinant sequences in different tissues makes highly unlikely that PCR-recombinants, if any, may have biased the results of the analysis.

**Figure 2:**
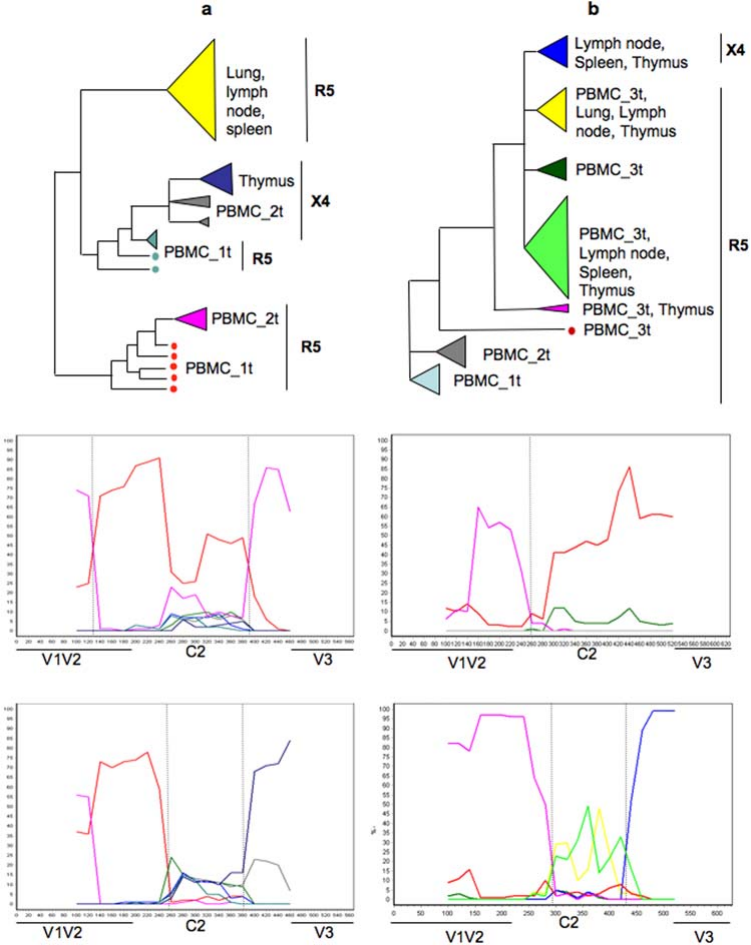
Recombination breakpoints in HIV-1 V1-V3 recombinant sequences from different subjects. The cladograms (top panels) represent the main non-recombinant lineages (putative parental sequences) within the maximum likelihood trees of subject S2 and S4. The bootscanning plots (middle and bottom panel) were obtained for representative HIV-1 V1-V3 recombinant sequences (query sequences). Horizontal axis indicates the nucleotide position along a query sequence; vertical axis gives the bootstrap support for the clustering of the query sequence and the parental sequence (or clade) with a matching color in the cladogram. Vertical dotted lines localize potential recombination breakpoints. a Subject S2. Middle panel: bootscanning plot of an R5 recombinant sequence from early PBMC (PBMC_1t). Bottom panel: bootscanning plot of an X4 recombinant sequence from early PBMC (PBMC_1t). b. Subject S4. Middle panel: bootscanning plot of an R5 recombinant sequence from late PBMC (PBMC_3t). Bottom panel: bootscanning plot of an X4 recombinant sequence from thymus.

Recombination breakpoints were mapped by bootscanning. In all 53 recombinant sequences, putative breakpoints were localized within the C2 domain, while no recombination was found within V1-V2 (data not shown). Representative bootscannings of two R5 and two X4 sequences are shown in Figure 2. R5 recombinants originated from ancestral sequences in peripheral blood, while the X4 recombinants combined R5 V1-V2 sequences from PBMCs and X4 V3 sequences from the thymus clade (subject S2), or the thymus/spleen/lymph clade (subject S4). Bootscanning plots for all other X4 recombinant sequences showed the same pattern (data not shown).

### Tempo and mode of R5 and X4 variants evolution during infection

**Figure 3:**
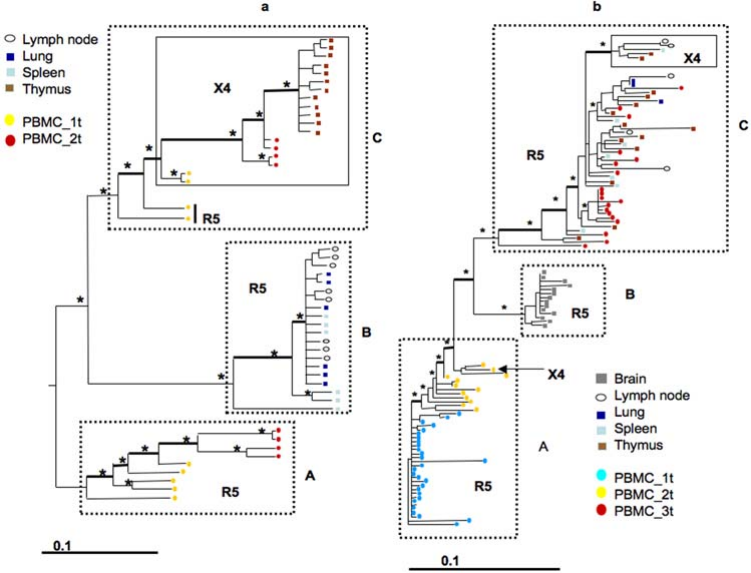
Maximum likelihood trees of HIV-1 V1-V3 sequences from longitudinal PBMCs and *post mortem* tissues of different subjects. Branch lengths were estimated with the HKY+Γ model and drawn in scale with the bar at the bottom indicating 0.1 nucleotide substitutions per site. The trees were rooted by maximum likelihood enforcing a molecular clock and taking into account different sampling dates. One * along a branch represent significant statistical support for the clade subtending that branch (zero-branch-length test *p*<0.001; Bayesian posterior >0.75, and/or bootstrap value >70%). Colored boxes and circles represent different tissues according to the color table to the left of each tree. Internal thick edges indicate population bottlenecks a. Patient S2. PBMC 1t and 2t were sampled 15 and 22 months after birth, respectively. Other tissues were sampled *post mortem* 26 months after birth. The main statistically supported clades, designated A, B, and C are highlighted within broken boxes. The solid box within clade C highlights X4 sequences. b. Patient S4. PBMC 1t, 2t, and 3t were sampled 3, 22, and 77 months after birth, respectively. The other tissues were sampled *post mortem* 77 months after birth. The main statistically supported clades, designated A, B, and C are highlighted by broken boxes. The solid box within clade C highlights X4 sequences.

Subjects S2 and S4 harbored X4 variants both in PBMCs and the thymus, and were selected for an in depth study of *in vivo* evolution of R5 and X4 quasispecies. Non-recombinant sequences in PBMCs over the course of infection and from terminal tissues were combined for a high-resolution phylodynamic analysis [26], [38]. The genealogy of HIV-1 V1-V3 sequences, sampled over two years of infection from subject S2, showed three main lineages, A, B, and C (Figure 3a). Each lineage was well supported by >75% Bayesian posterior probability, *p* values≤0.001 in the zero branch length test, and >70% bootstrap. Moreover, both ML and Bayesian-based methods inferred the same root for the tree. Lineage A including the R5 viral sequences from early and late PBMCs displayed clear temporal structure. Strains from early PBMC samples passed through an initial population bottleneck, followed by a second bottleneck leading to the emergence of a new monophyletic subcluster that included sequences only from late PBMC samples. HIV-1 sequences from *post mortem* lung, spleen, and lymph nodes were exclusively R5, clustered as a separate phylogenetic lineage within clade B, and included at least two bottlenecks. Temporal structure was also evident in clade C where initial populations of R5 and X4 strains isolated from early PBMCs were replaced through a bottleneck by a subclade containing only X4 variants from early and late PBMCs and from the thymus. HIV-1 X4 strains isolated from late PBMCs emerged after a second bottleneck, while the last bottleneck gave rise to a subclade consisting exclusively of X4 viral strains from the thymus.

The inferred phylogeny for subject S4 was based on evaluation of sequences over a period of about 6.5 years and indicated at least three statistically supported clusters: A, B, and C (Figure 3b). All viral sequences from PBMCs at sampling time T1 and T2, as well as from the brain, displayed V3 loops predicted to use the R5 coreceptor. The only exception was the presence of an early X4 PBMC variant at time T2. As in subject S2, sequences of HIV-1 strains from S4 PBMCs at different time points were temporally structured: PBMCs sampled at time T1 clustered at the base of the tree, near to the root, and were replaced through a bottleneck by a new population from samples collected at later time points. Sequences from brain were exclusively R5 and belonged to a separate monophyletic clade (clade B in Figure 3b). Clade C included HIV-1 strains from PBMCs at T3 with contemporaneous variants in the lung, lymph nodes, spleen and thymus. In contrast, the monophyletic cluster at the top of the tree included only X4 HIV-1 variants that were found in thymus, lymph nodes, and spleen. Overall, the structure of the trees from both individuals suggested a gradual emergence from R5 to X4 sequences through continuous selection of new variants evolving over time followed by an expansion after the last bottleneck of the X4 population.

### *In vivo* evolutionary rates of R5 and X4 populations

To test the hypothesis that the expansion of the X4 population of viruses might be due to an increased replication rate that would accelerate evolution, molecular clock analysis was used to estimate the rate of evolution for R5 and for X4 variants. Mean evolutionary rates of R5 or X4 strains within subject S2 were not significantly different. The mean evolutionary rate of the R5 strains was 1.17×10^−2^ nucleotide substitutions per site per year (0.1–2.2×10^−2^ lower and higher 95% highest posterior density, HPD), while the rate for X4 strains was 1.6×10^−2^ nucleotide substitutions per site per year (0.7–2.6×10^−2^ lower and higher 95% HPD). Although absence of longitudinal X4 sequences within subject S4 precluded estimation of evolutionary rates for X4 subpopulations, an evolutionary rate of 2.4×10^−2^ nucleotide substitutions per site per year (1.2–3.7×10^−2^ lower and higher 95% HPD) was estimated for the S4 R5 strains in PBMCs. Evolutionary rate for R5 strains in subject S4 were not significantly different (*p*<0.001) from the evolutionary rate of either the R5 or the X4 strains within subject S2.

### Selection analysis during population bottlenecks

**Figure 4:**
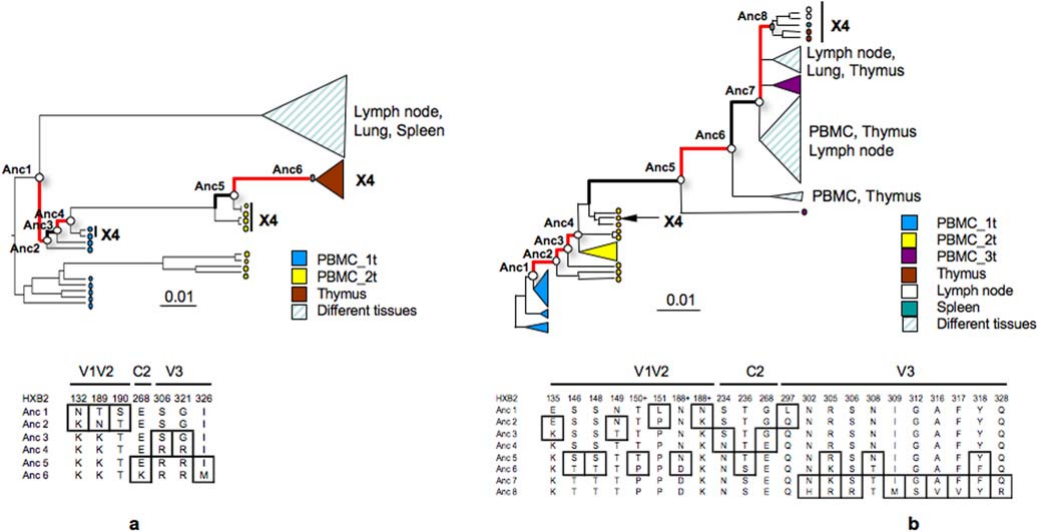
Selection pressure in HIV-1 V1-V3 during intra-patient viral population bottlenecks. Rooted Bayesian phylogenetic trees were obtained using the SRD06 relaxed clock model. Branch lengths were drawn in scale with the bar at the bottom indicating 0.01 nucleotide substitutions per site. Triangles represent clades for which statistically unsupported internal branches were collapsed. Ancestral (Anc) sequences were reconstructed by maximum likelihood. Internal branches under significant positive (dN/dS>2) or negative (dN/dS<0.05) selection are drawn in thick red and black lines, respectively. Open circles emphasize the parent (ancestral sequence before the bottleneck) and child (ancestral sequence following the bottleneck) nodes. Specific amino acid positions (according to the HXB2 HIV-1 reference sequence) under positive selection within ancestral sequences are given in the tables at the bottom of each tree. Sequences with predicted X4 coreceptor use are indicated. a. Subject S2. Longitudinal PBMC and *post mortem* tissue samples were the same used for the tree in Figure 2a. b. Subject S4. Longitudinal PBMC and *post mortem* tissue samples were the same, with the exclusion of sequences from brain, used for the tree in Figure 2b.

Multiple viral population bottlenecks within R5 strains preceded the bottleneck leading to appearance of X4 variants in both individuals (Figure 4). To investigate the evolutionary driving forces at work during such bottlenecks, we performed a ML-based selection pressure analysis of the internal branches in the S2 and S4 genealogy (Figure 4a and 4b). In each case, the best fitting model was the one that allowed for both positive and negative selection along the internal branches involved in the bottlenecks (p<0.05). Estimated dN/dS ratios were greater than 2 (positive selection) or less than 0.5 (purifying selection) along the internal branches. In contrast, dN/dS values were not significantly different from 1 along the internal branches of the clade that included the R5 HIV-1 quasispecies from the brain of patient S4. HIV-1 evolutionary dynamics appeared remarkably similar within both subjects. In general, bottlenecks driven by positive selection were usually followed by a bottleneck driven by purifying selection.

### Genotypic changes associated with selection

To identify amino acid replacements most likely involved in the adaptive response of the viral quasispecies to selection pressure, V1-V3 ancestral sequences involved in the major bottlenecks from patient S2 and S4 were inferred (Figure 4). A similar evolutionary pattern appeared to underlie the gradual development of X4 variants from an initial R5 population in both subjects (the full V1-V3 alignment of the ancestral sequences from both subjects is given in supplemental Figure S1). Sites under positive selection generally occurred within the N-terminal portion of V1, within amino acid positions 132-151, and the C-terminal portion of V2, within positions 188-190, while the few sites under positive selection in C2 were interspersed along the domain. V3 mutations under positive selection were distributed across the domain and often involved replacements with high-charged basic amino acids along the branches leading to X4 variants. Generally, positively selected substitutions in V1, V2, and C2 appeared along the earlier branches of the genealogies (between Anc1/Anc2 of subject S2 or between Anc1/Anc2, Anc2/Anc3, Anc3/Anc4 of subject S4), and were fixed in all subsequent viral populations. Selected substitutions in V3 appeared only after V1-V2 changes along the late bottlenecks (between Anc3/Anc4 and Anc5/Anc6 of S2 or between Anc5/Anc6 and Anc7/Anc8 of S4). The mutation from serine to arginine at position 306, which is associated with coreceptor use [47], appeared in the ancestral sequences at the origin of X4 lineages (between Anc5/Anc6 in S2 and Anc7/Anc8 in S4). Position 268 in C2 was also under positive selection in both subjects, although in one case G268E appeared during an early bottleneck within the R5 population (subject S4), while in a second case, E268K appeared during a late bottleneck within the X4 population (subject S2).

### Migration analysis

**Figure 5:**
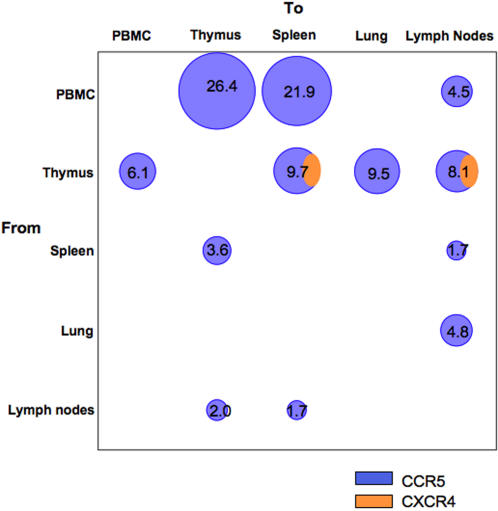
HIV-1 migration analysis among different tissues for patient S4. Each circle is proportional to the percentage of observed migrations (given within the circle) inferred from the S4 maximum likelihood cladogram in Supplemental Figure s2. Migration counts less than 1% were not indicated.

While viral sequences from the thymus of subject S2 were X4, mixtures of R5 and X4 quasispecies were found in the thymus and other lymphoid organs from subject S4. To assess the HIV-1 population dynamic within patient S4, the direction of gene flow among *virodemes* in late PBMC samples and *post-mortem* tissues was tracked (Figure 5). Sequences from the brain represented a separate compartment of R5 strains [21]–[26], and were not included in the gene-flow analysis. Migration events among different tissues were significantly less than those expected from a random model in which each *virodeme* is freely diffusible and equally likely to exchange virus with any other one (*p*<0.0001). Results supported a model of restricted gene-flow within viral subpopulations in the thymus and other tissues (Figure 5). PBMCs and thymus accounted for about 86% of total HIV-1 gene outflow, with 53% from PBMCs (R5 sequences) and 33% from thymus (both R5 and X4 sequences).

## Discussion

Studies of HIV-1 evolution *in vivo* have focused primarily on a “whole body” approach, where viral evolution is mainly inferred from cell-free or cell-associated HIV-1 genomes in blood or, occasionally, within one or two tissues [3], [19]–[21], [23], [24], [26]. In contrast, our study included detailed mapping of the evolutionary patterns of HIV-1 *virodemes* in blood, as well as lymphoid and non-lymphoid tissues, and applied phylogenetic and population genetic tools to examine the dynamics of virus interaction within the host. Most studies have focused almost exclusively on the V3 loop as the genetic marker, while none tested for positive selection in the internal branches of reconstructed genealogies, which is a hallmark of ancestral episodic selection leading to adaptive response [48]. Inclusion of *env* V1-V2 domains coupled with internal branch tests for positive selection in our analysis was critical to uncover episodic selection within HIV-1 quasispecies.

The role of selection and random genetic drift in the *in vivo* evolution of HIV-1 envelope and in the emergence of CXCR4 variants associated with rapid disease progression has been debated for some time with some evidence supporting each model [3], [49], [50]. In our study, positive selection involving amino acid residues in the V1, V2 and C2 domains was detected before the emergence of X4 strains, i.e. independent of coreceptor switch. In contrast, the ancestral sequences at the origin of the X4 strains in the thymus and other lymphoid organs contained amino acid replacements in V3 leading to increase net charge of the V3 loops.

The natural history of HIV-1 among the infected subjects seemed to be defined by sequential population bottlenecks characterized by temporally ordered patterns of amino acid substitutions. Within each individual X4 variants evolved *de novo* from R5 ancestors ruling out the hypothesis of long term sequestration of transmitted X4 variants. Although, in general, different spectra of amino acid replacements in the V1-C2 gp120 domains developed in each individual, sites under positive selection in early bottlenecks were generally restricted within the N-terminal portion of V1 and the C-terminal portion of V2. Three positions in V2, C2, and V3, were found to be under positive selection across subjects. Substitutions that accumulated in specific amino acid residues *in vivo* were identical to amino acid changes that developed during *in vitro* evolution [14]. *In vitro*, combinations of V3 substitutions can lead to major loss of entry fitness or even lethality unless compensated by mutations in or near V1-V2 [14], [51]. Our study provides evidence that changes outside the V3 domain may be essential for setting the background for the emergence HIV-1 X4 strains *in vivo*, in agreement with reports indicating that other Env regions outside V3 contribute to CXCR4 coreceptor use and cell tropism [9], [14]–[16]. Overall, our data suggest that the evolution of HIV-1 envelope involved a complex, but nonetheless ordered and potentially restricted, developmental program that was recapitulated in different individuals.

In addition to amino acid substitutions in V1-C2, recombination was detected between V1-V2 and V3 in two individuals. Rather than random distribution of putative breakpoints in the recombinants, essentially all sequences had crossover localized to the C2 region. Evolution of HIV-1 in the brain of one subject was highly compartmentalized and limited to CCR5-using variants, in agreement with previous findings [26], [52]. An elevated recombination rate among the R5 sequences in the brain, which our data identified, would be consistent with a long independent evolution of a segregated viral subpopulation in a separate compartment. Since only *post mortem* samples were available from brain tissue, phylogenetic analysis could not exclude earlier input of viral sequences and turnover similar to PBMCs. Analysis of X4 strains of recombinant origin showed that recombination always occurred within C2 between ancestral V1V2 sequences of R5 phenotype from PBMCs and ancestral V3 sequences of X4 phenotype from thymus, suggesting that the thymus may play an important role in the amplification of CXCR4-using strains.

We also found evidence of bottlenecks characterized by strong purifying selection, after a positive selection episode, suggesting the presence of temporary adaptive peaks in the fitness landscape. In fact, it is expected that if a population reaches a local high-fitness peak, most of the variants in the next generation would be removed by purifying selection or genetic drift. Emergence of fitter HIV-1 strains through the bottlenecks could be due to multiple selective factors: antiretroviral therapy, cellular immune control, change in target cell populations or change in host milieu favoring X4 variants [53], and/or emergence of viral variants with enhanced entry efficiency. In the subjects in our study, positive selection occurred in the absence of combination antiretroviral treatment, ruling out drug selective pressure. All patients had severely suppressed CD4 T cells and none of the known B-cell epitopes (listed at the Los Alamos HIV databases) were localized in the V1-V3 region of sequences from any of our subjects, arguing against immune control as a major selection factor, although lack of immunity, as found in the SIV model, may provide a selective advantage [54]. Our data are consistent with a model of enhanced viral entry efficiency combined with host milieu as major selective pressures driving episodic selection.

The results obtained in the present work also point to the potential importance of the thymus for the evolution/amplification of the X4 coreceptor use. Thymocytes express high levels of CD4 and CXCR4 and a transcriptionally active environment that promotes viral replication [55]. Trafficking of lymphocytes between thymus and secondary lymphoid tissues is highly regulated and typically unidirectional [56]. The finding in one of the subjects that HIV-1 X4 gene flow was from thymus to lymphoid tissues, but not *vice versa*, is consistent with trafficking patterns of thymocytes and implicates X4-infected thymocytes as a potential mechanism for systemic dissemination of X4 variants. Studies based on experimental observations and mathematical models suggest that the increased turnover rate of naïve T-cells over time in HIV-1 infected patients could be a consequence of progressive depletion of memory T-cells in the periphery, and may explain the increased fitness of X4 viruses and their emergence in about 50% of the individuals during the late stage of the disease [57]. Our finding that the evolutionary rates of R5 and X4 HIV-1 sub-populations are not significantly different support the hypothesis that amplification of X4 variants is due to factors other than an increased rate of evolution and may be linked to the availability of target cells. In contrast to early reports that thymic function declines during adolescence and almost disappears in early adulthood [58], a substantial body of evidence now shows that the adult thymus retains some thymopoietic function and continues to produce naïve T-cells for export to the periphery [59]–[62], raising the possibility that our findings from HIV-1 infected children may be applicable to older individuals, as well.

Some limitations of the present study should be recognized. We examined HIV-1 quasispecies in thymus tissues from only four subjects. Since no *ante mortem* thymic or lymphoid tissues were available, the exact evolutionary history of the X4 population in the thymus at earlier time points is uncertain. For example, we cannot exclude that similar X4 variants may have emerged in other tissues (such as lymph nodes or spleen) prior to or concomitant with their appearance in the thymus. Also, the result of the migration analysis is not informative about the viral flow during early infection and would require further investigation using longitudinal samples from different subjects to be confirmed. However, while it is feasible to collect serial PBMC samples from earlier time points, serial biopsies of tissues such as thymus and spleen are hardly an option in human patients. Such a difficulty points to the importance of animal models, such as SIV-infected rhesus macaque, to discern the role for the thymus and secondary lymphoid tissues in evolution of X4 variants.

Further clarification of the key-role of the thymus and other lymphoid tissues in the evolution/amplification of X4 strains might have important consequences for the development of effective therapeutic strategies. R5 coreceptor-blocking agents may be extremely efficient, during the early stage of the disease, in avoiding the emergence of the X4 quasispecies associated with certain types of disease progression. On the other hand, the development of X4 entry inhibitors and/or drugs able to target the export of X4 infected T-cells from the thymus could be critical to control HIV-1 infection in advanced disease stages. If breakdown of immunity contributes to selection for X4 variants, then immune-based strategies may delay or prevent amplification of X4-using viruses independent of the tissue of origin. A detailed mapping of V1-V3 sites under positive selection associated with increased viral entry efficiency will lay the foundations for the development and evaluation of such novel drugs, and our study has shown the potential power of phylodynamics and high-resolution phylogeny for accomplishing this important task.

## Materials and Methods

### Subjects and samples

Four pediatric subjects infected by maternal HIV-1 transmission (S1, S2, and S4) or by neonatal blood transfusion (S3) were enrolled under a protocol approved by the Institutional Review Board of the University of Florida, College of Medicine. The mothers of the children enrolled in the study has already given consent for the collection and storage of blood and tissue samples and for collection of clinical data as part of the protocol implemented in Dr. Goodenow's lab entitled: Biological implications of HIV-1 genetic variability. All patients developed AIDS before one year of age, and died of AIDS-related illnesses by 8 months (S1), 26 months (S2), 6.5 years (S4), or 7.5 years (S3) of age. Subjects received antiretroviral therapy with nucleoside reverse transcriptase inhibitors (NRTI), but no combination therapy with non-NRTI or protease inhibitors. Tissues including lung, mesenteric lymph nodes, spleen, thymus, and brain were obtained *post mortem*, while peripheral blood mononuclear cells (PBMCs) were obtained at or near the time of death for all subjects and over the course of infection for S2, and S4. In patient S4 brain tissues were mostly sampled from the frontal lobe. DNA was extracted from cryopreserved PBMC samples as previously described [63], [64]. Tissues were quick frozen in liquid nitrogen in 50 ml conical tubes and stored at −80°C until processed for DNA extraction. DNA was extracted from multiple biopsies from each tissue using the Dneasy tissue extraction kit (QIAGEN, Valencia, CA) [65]. Several DNA extractions from each tissue were pooled together, and multiple PCR amplifications were performed on the combined DNA extraction to ensure representation of viral sequences within a tissue.

### Amplification, cloning and sequencing

The V1-V3 hypervariable region of envelope was amplified using primers and conditions previously described [66], followed by ligation into PCR 2.1 vector (Invitrogen, Carlsbad, CA) and transformation of competent Top10F/(Invitrogen, Carlsbad, CA) cells. Sequences were prepared with DYEnamic ET dye terminator cycle sequencing kit for MegaBACE DNA Analysis Systems (GE Healthcare, Chalfont St. Giles, United Kingdom), and run on a MegaBACE 1000 (GE Healthcare) in the Genome Sequencing Service Laboratory at the University of Florida.

### Analysis of V1-V3 sequences and coreceptor usage prediction

Sequences were edited, verified, and entered into HIV_base_ for retrieval and analysis [67]. For each domain an amino acid alignment was obtained manually using our motif-base alignment method [68] and translated back to nucleotides for further analysis. HIV-1 subtype was assessed with the Rega HIV subtypying tool version 2.0 (http://www.bioafrica.net/virus-genotype/html/subtypinghiv.html). V1-V3 sequences from all subjects clustered with subtype B reference sequences. Coreceptor usage was predicted with two different algorithms: 1. By calculating the net charge of the V3 loop based on number and position of amino acid residues (K+R)-(D+E) [39]; 2. By using a position-specific scoring matrix (PSSM) developed for subtype B sequences [40], [41]. Both methods gave the same results except for three sequences for which coreceptor usage had to be determined experimentally (see below).

### Determination of coreceptor usage and actual phenotype

The V1-V5 hypervariable region of envelope of each strain was amplified as described previously [66]. V1-V5 sequences were cloned into pcDNA expression vectors and used to generate single-cycle viruses tagged with luciferase (luc), as previously described [9], [66]. To determine coreceptor use and cell tropism, PBMC, monocyte-derived macrophages, and MT-2 cells were infected with V1-V5 single-cycle *env* pseudo-typed viruses in the absence or presence of monoclonal antibody specific for either CCR5 (2D7) or CXCR4 (12G5) (AIDS Research and Reference Reagent program, Division of AIDS, NIAID, NIH). Virus phenotype was defined [69].

### Recombination analysis

HIV-1 gp120 *env* sequences (V1 to V3 domains) from all individuals were evaluated in a single phylogenetic tree that verified the integrity of the data. Separate phylogenetic trees for V1-V2 and C2-V3 domains were also obtained to detect putative recombinant sequences that may cluster differently in different trees. The presence of recombination was confirmed with the PHI test, which is based on the notion of refined incompatibility score [70], and it is implemented in SplitsTree package version 4.8 [71]. Extensive simulation studies and comparison with other available methods have shown that not only the PHI test is extremely powerful in detecting recombination, but it is also the method producing the lowest number of false positives [70]. Significance of the PHI statistic for the presence of recombination is assessed with the normal approximation of a permutation test where, under the null hypothesis of no recombination, sites along the alignment are randomly permuted to obtain the null distribution of PHI: *p*<0.05 indicate significant presence of recombination [69].

### Mapping recombination breakpoints

Recombinant sequences were analyzed with the bootscanning method implemented in the Simplot package [72] to locate putative recombination breakpoints. Bootscanning infers phylogenetic trees using a sliding window along an alignment including a query sequence (the putative recombinant sequence) and putative parental (non-recombinant) sequences. For each tree along the alignment 1000 bootstrap replicates are generated and the bootstrap support for the clustering of the query sequence with each of the pre-defined parental groups is recorded. Bootscanning plots, like the ones showed in Figure 3, display how the bootstrap support (y-axis) for the clustering of the query sequence with the parental sequences changes along the alignment (x-axis). Since recombination leads to the creation of mosaic genomes originated from different ancestors, a recombination event can be detected by the “jumping” of the query sequence between highly supported phylogenetic clades in trees obtained from different genomic regions [73]. Bootscanning plots were obtained using a window of 200 nucleotides sliding forward in steps of 20 nucleotides. Trees were inferred by NJ using HKY estimated distances with a transition transversion ratio empirically estimated by maximum likelihood for each window.

### Phylogenetic Analysis of non-recombinant data sets

A total of 33 sequences for S1 (median per tissue 8, range 5 to 13); 49 sequences for S2 (median/tissue 9, range 6 to 12); 17 sequences for S3 (median/tissue 8, range 1 to 17); and 103 sequences for S4 (median/tissue 15, range 3 to 35) were analyzed. The best fitting nucleotide substitution model was tested with a hierarchical likelihood ratio test, using a neighbor-joining (NJ) tree with LogDet corrected distances [74]. Maximum likelihood (ML) trees were then inferred with the selected model (HKY+Γ, 8 categories, for all data sets) and ML-estimated substitution parameters. The heuristic search for the best tree was performed using an NJ tree as starting tree and the TBR branch-swapping algorithm. Neighbor-Joining (NJ) trees were also estimated using pair-wise distances inferred by ML with the best fitting nucleotide substitution model. Calculations were performed with PAUP* 4.0b10 written by David L. Swofford. Statistical support for internal branches in the NJ trees was obtained by bootstrapping (1000 replicates) for the NJ trees and the ML-based zero branch length test for the ML trees [74]. Trees were rooted by ML rooting by selecting the rooted tree with the best likelihood under the molecular clock constraint, or by outgroup rooting using the earliest PBMC samples as outgroup. The location of the root was confirmed by inferring rooted Bayesian trees with a relaxed clock model and exponentially distributed evolutionary rates as prior. The Bayesian calculation consisted of 50,000,000 generations Markov Chains Monte Carlo (MCMC) with sampling every 5000^th^ generation using the BEAST software package version 1.4 (http://evolve.zoo.ox.ac.uk/beast/). Convergence of the MCMC was assessed by calculating the effective sampling size (ESS) of the combined runs [75]. All parameter estimates showed significant ESS (>250). Bayesian trees were also obtained with the program MrBayes v3.1.2, using the HKY+Γ model, running in parallel two MCMC for 10,000,000 generations with sampling every 100^th^ generation. Convergence was assessed by comparing the average standard deviation of split frequencies (*p*<0.0001). Statistical support for each clade in the Bayesian trees was obtained by calculating clade-specific Bayesian posterior probabilities with MrBayes. In each case, ML and Bayesian methods inferred the same topology.

### Reconstruction of ancestral sequences and positive selection analysis

Ancestral amino acid sequences in the genealogy obtained for each patient were inferred by the maximum likelihood method, using the codon substitution model M0 [76]. Positive selection analysis was performed by comparing the different *Branch* maximum likelihood codon substitution models using the improved test 2, which tests for different dN/dS (nonsynonymous/synonymous substitutions) ratios along given branches of the tree [76], [77]. Three different models were compared: model 0, assuming a single dN/dS for the entire tree; model 1, assuming a different dN/dS for each branch in the tree, and model 2 assuming a baseline dN/dS = 1 for the entire tree and several different dN/dS along the branches of the major bottleneck events in the genealogy. Model 0, model 1, and model 2 are nested and a hierarchical likelihood ratio test was used to check which model fitted the data significantly better [76], [77]. Average dN/dS ratios among maximum likelihood inferred ancestral sequences at the internal nodes of the significantly supported monophyletic clades of the trees were also compared. Specific amino acid changes along the internal branches of the tree were inferred by maximum likelihood. Calculations were performed with the PAML package [76].

### Molecular clock analysis

Ultrametric trees were obtained by enforcing a molecular clock on the inferred genealogy, and re-estimating the branch lengths and substitution parameters with maximum likelihood with the previously selected evolutionary model. The clock hypothesis was tested with the likelihood ratio test. Evolutionary rates with a strict and a relaxed clock model using a lognormal distribution as prior [75] were obtained with the Bayesian framework implemented in the BEAST program version 1.4, by running 50,000,000 Markov Chain Monte Carlo MCMC with sampling every 5000^th^ generation. Calculating the ESS assessed convergence of the MCMC procedure. ESS>250 were considered significant.

### Gene flow tests and migration counts

The hypothesis of compartmentalization, i.e. the existence of distinct HIV-1 sub-populations in different tissues, was tested by the Slatkin and Maddison test for gene flow [78] using the MacClade version 4 program (Sinauer Associates, Sunderland, MA). A one-character data matrix is obtained from the original data set by assigning to each *taxon* in the tree a one-letter code indicating its tissue of origin. Then, the phylogenetic tree obtained from the nucleotide aligniment is imported in MacClade and the putative origin of each ancestral sequence (i.e. internal node) in the tree is inferred with the Fitch algorithm [79] by finding the most parsimonious reconstruction (MPR) of the ancestral character (this can be accomplished by selecting the *Trace character* option in the *Trace* menu of MacClade). The result is a tree colored as in supplemental Figure S2, were each colored branch represent the tissue of origin of the internal node (ancestral sequence) or tip node (actual sequence) subtending that branch. A change in color (i.e. in tissue assignment) between two branches connected trough a node represent a migration event from one tissue to another that must have occurred during the genealogical evolution of the sequences under investigation. The final tree-length, i.e. the number of observed migrations in the genealogy, computed by MacClade can be compared to the tree-length distribution of 10,000 trees obtained by random joining-splitting (by selecting the *Character steps/etc* option from the *Char* menu). Observed genealogies significantly shorter than random trees indicate the presence of subdivided populations [78]. Specific migrations among different compartments (states) were traced with the *State changes and stasis* tool (MacClade), which counts the number of changes in a tree for each pair-wise state. When multiple MPRs were present (as in our data sets), the algorithm calculated the average migration count over all possible MPRs for each pair. The resulting pair-wise migration matrix is then normalized to obtain the percentage of observed migration to/from different tissues in the tree.

## Supporting Information

Table S1 Distribution of HIV-1 V1V3 recombinant sequences
(0.03 MB DOC)

Figure S1 V1-V3 multiple alignment of reconstructed ancestral sequences from patients S2 and S4. Ancestral (Anc) sequences, corresponding to the internal nodes in the trees in Figure 4, are aligned with the HIV-1 reference strain HXB2 on top. Blank spaces indicate gaps. A dash indicates that the amino acid matches the corresponding position of HXB2. In the V3 loop, red and blue shadings indicate positively and negatively charged amino acids, respectively, which are important for the determination of coreceptor use. Two arrows indicate residues 11 and 25 considered important for coreceptor use (Fouchier et al. 1994). Specific sites under positive selection are outlined by solid boxes.
(0.24 MB TIF)

Figure S2 Maximum parsimony migration analysis for patient S4. Cladogram of HIV-1 sequences from different tissues showing clade C of the maximum likelihood tree given in Figure 3. The color of a branch indicates the tissue of origin of the top node (ancestral sequence) or tip (actual sequence) of that branch according to the color table in the figure. Tissues of origin for the internal nodes (ancestral sequences) of the cladogram were inferred with the Fitch (1971) algorithm.
(0.28 MB TIF)
