# Supplementary material for: Phylodynamics of HIV-1 in Lymphoid and Non-Lymphoid Tissues Reveals a Central Role for the Thymus in Emergence of CXCR4-Using Quasispecies
Source: PLoS One. 2007 Sep 26;2(9):e950. doi: 10.1371/journal.pone.0000950 (PMC1978532; doi:10.1371/journal.pone.0000950)
Supplement: Table S1 — Distribution of HIV-1 V1V3 recombinant sequences (0.03 MB DOC) [file pone.0000950.s001.doc]

Supplemental Table s1. Distribution of HIV-1 V1V3 recombinant sequences

| Subject | Recombinant sequences | Tissue of origin | R5 (number of sequences) | X4 (number of sequences) |
| --- | --- | --- | --- | --- |
| S2 | 10.9% (6/55) | PBMC  Lung  Lymph node | 1  3  1 | 1  -  - |
| S4 | 31.3% (47/150) | PBMC  Spleen  Thymus  Brain | 5  1  2  31 | -  3  5  - |
