# Supplementary figures and images for: Phylodynamics of HIV-1 in Lymphoid and Non-Lymphoid Tissues Reveals a Central Role for the Thymus in Emergence of CXCR4-Using Quasispecies
Source: PLoS One. 2007 Sep 26;2(9):e950. doi: 10.1371/journal.pone.0000950 (PMC1978532; doi:10.1371/journal.pone.0000950)

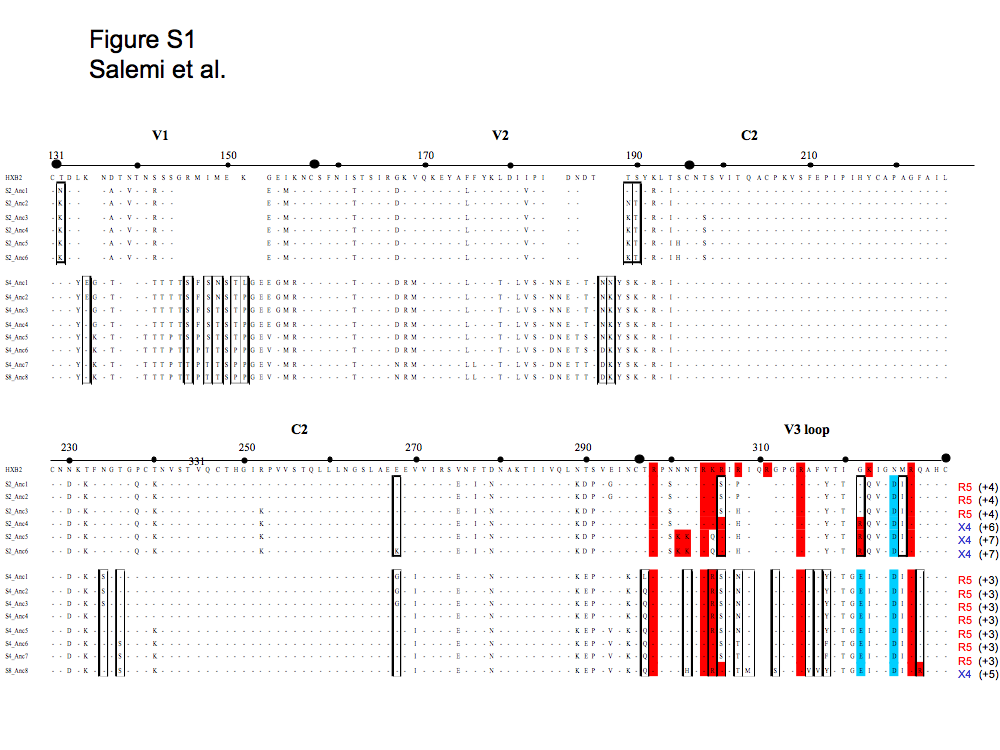

Supplement: Figure S1 — V1-V3 multiple alignment of reconstructed ancestral sequences from patients S2 and S4. Ancestral (Anc) sequences, corresponding to the internal nodes in the trees in Figure 4, are aligned with the HIV-1 reference strain HXB2 on top. Blank spaces indicate gaps. A dash indicates that the amino acid matches the corresponding position of HXB2. In the V3 loop, red and blue shadings indicate positively and negatively charged amino acids, respectively, which are important for the determination of coreceptor use. Two arrows indicate residues 11 and 25 considered important for coreceptor use (Fouchier et al. 1994). Specific sites under positive selection are outlined by solid boxes. (0.24 MB TIF) [file pone.0000950.s002.tif]

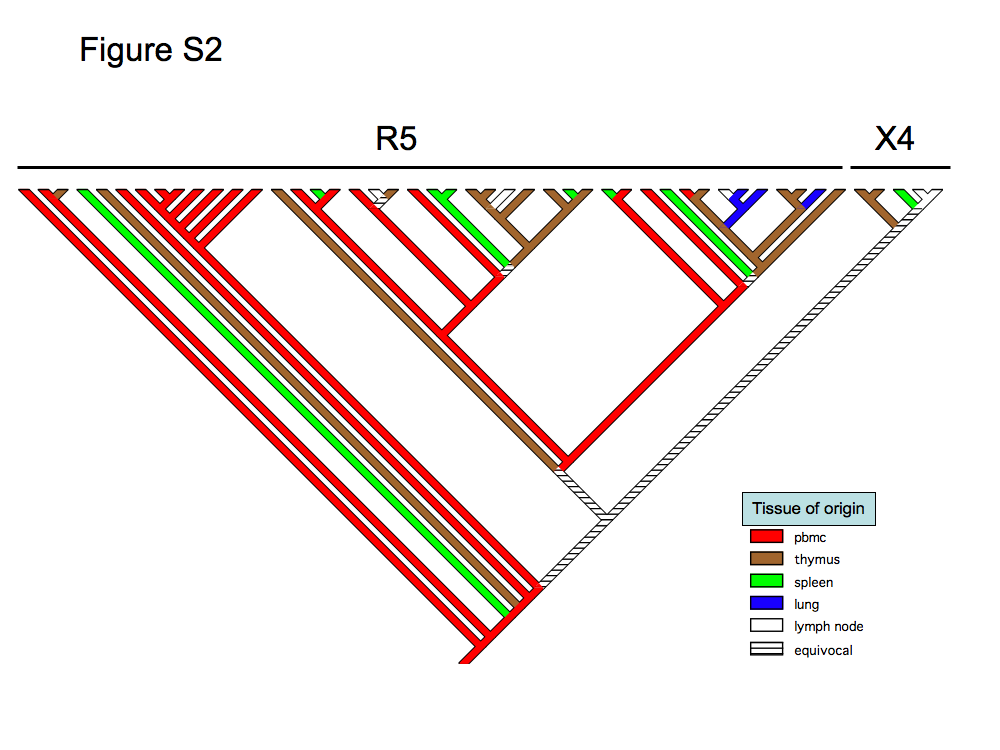

Supplement: Figure S2 — Maximum parsimony migration analysis for patient S4. Cladogram of HIV-1 sequences from different tissues showing clade C of the maximum likelihood tree given in Figure 3. The color of a branch indicates the tissue of origin of the top node (ancestral sequence) or tip (actual sequence) of that branch according to the color table in the figure. Tissues of origin for the internal nodes (ancestral sequences) of the cladogram were inferred with the Fitch (1971) algorithm. (0.28 MB TIF) [file pone.0000950.s003.tif]
